# A Sandwich-Type Impedimetric Immunosensor for the Detection of Tau-441 Biomarker

**DOI:** 10.3390/bioengineering12080805

**Published:** 2025-07-27

**Authors:** Khouloud Djebbi, Yang Xiang, Biao Shi, Lyes Douadji, Xiaohan Chen, Jin Liu, Chaker Tlili, Deqiang Wang

**Affiliations:** 1 Chongqing Institute of Green and Intelligent Technology, Chinese Academy of Sciences, Chongqing 400714, China; djebbi93khouloud@gmail.com (K.D.); xy@cigit.ac.cn (Y.X.); shibiao@cigit.ac.cn (B.S.); ldouadji@cigit.ac.cn (L.D.); chenxiaohan@cigit.ac.cn (X.C.); dqwang@cigit.ac.cn (D.W.); 2 University of Chinese Academy of Sciences, Beijing 400714, China; 3 School of Mechanical and Vehicle Engineering, Chongqing University, Chongqing 400044, China; 4 Department of Pathology, Chongqing General Hospital, Chongqing 401147, China

**Keywords:** Alzheimer’s disease, Tau-441, self-assembled monolayers (SAMs), sandwich-based assay, electrochemical impedance spectroscopy (EIS)

## Abstract

The human Tau protein stands for one of the most conspicuous and crucial hallmarks of Alzheimer’s disease (AD) diagnosis, along with other tauopathies. However, the assay for direct detection of tiny Tau protein concentrations in human samples continues to pose a significant challenge for the early diagnosis of AD. Thus, an amplification-based strategy is required. In this proposed work, we established an impedimetric immunosensor to detect human Tau-441 protein in PBS buffer using a sandwich approach, wherein we employed two distinct monoclonal antibodies (HT7 and BT2) that specifically recognize the amino acids 159–198 of the target protein. Through this strategy, we were able to detect as low as 0.08 pg/mL. These findings were attributed to the use of a biotinylated antibody (BT2)-streptavidin complex, which facilitated the amplification of the normalized signal, resulting in a lower limit of detection in comparison to the directly based immunosensors. Subsequently, we investigated the designed immunosensor to assess the assay’s selectivity in the presence of different off-targets, and no cross-interaction was recorded. The outcomes of our study provide valuable new insights into the application of sandwich-based assay as a highly sensitive and selective immunosensor for the detection of small protein.

## 1. Introduction

Alzheimer’s disease (AD) represents a neurodegenerative disorder initially identified by Alois Alzheimer in 1906 [1]. According to the World Alzheimer Report 2024, AD is the most common cause of dementia, accounting for an estimated 60% to 70% of cases, with approximately 55 million people estimated to have dementia across the world, and the number is expected to reach 139 million by 2050 [2]. Furthermore, the annual cost associated with dementia was estimated at USD 1.3 trillion in 2019, with expectations to exceed USD 2.8 trillion by 2030 [2]. AD is characterized by a dramatic decline of the cerebral cortex, reduced brain cell size, and the presence of fatty deposits within the blood vessels. A number of studies have indicated that initial symptoms of this malady can manifest 10 to 20 years prior to the onset of clinical symptoms [3]. Different techniques have been established for AD monitoring, such as neuro-imaging methods [4,5]; however, these methods tend to be costly, require high-tech equipment, and often lack the required sensitivity and selectivity. Moreover, due to the emergence of Alzheimer’s disease and the scarcity of ultimate treatment, early diagnosis is compulsory to avert its progression and boost the treatment outcomes. Thus, it is crucial to develop a sensitive and selective approach for its detection.

Currently, numerous biomarkers offer new opportunities to promote early disease diagnosis [4]. Biomarkers represent quantifiable indicators secreted during a specific stage of the malady, helping in the diagnosis and assessment of the treatment response [5]. The primary biomarkers thoroughly investigated for Alzheimer’s disease include extracellular amyloid beta-peptide (Aβ) plaques and intracellular neurofibrillary tangles (NFTs) [6]. Since their discovery, the diagnosis of AD has advanced significantly due to the association with increased levels of both total and phosphorylated Tau protein, alongside a decrease in amyloid peptide levels in the cerebrospinal fluid (CSF) samples [7]. The Tau protein, with a molecular weight of 45–65 kDa, belongs to the microtubule-associated protein (MAP) family [8]. It is predominantly located in neuronal cells, where it stabilizes the microtubules, thereby facilitating the transport and uptake of nutrients, and it contributes to the maintenance of neuronal integrity. The hyper-phosphorylation of Tau protein via protein kinases is assumed to stimulate its aggregation and the de-polymerization of microtubules. As a result, Tau tangles are generated during the aggregation event, and the microtubule structure cooperates with cell integrity, leading to neurodegeneration and cell-to-cell death [9].

Over the past decade, researchers have reported numerous strategies for detecting and identifying Tau protein. These methods were primarily based on enzyme-linked immunosorbent assays [10] and optical immunoassays [11], which rely on the interaction between the epitope of a monoclonal or polyclonal antibody and its corresponding binding site on the antigen. These conventional techniques employed are typically characterized by their high selectivity and sensitivity. However, their major drawbacks include not only the extended processing time but also their high costs, along with the necessity for highly skilled personnel or high-tech equipment, making them unsuitable for field applications. During the past few years, numerous biosensors have been developed as an alternative to the conventional methods for detecting Tau proteins. These biosensors embrace surface plasmon resonance (SPR) fiber sensors [12], surface-enhanced Raman scattering (SERS) spectroscopy [13], and fluorescent-based immunosensors [14]. Nonetheless, they display tremendous sensitivity and selectivity, a rapid time-to-result rate, and outstanding applicability for clinical or biological tests. The use of costly equipment limits their implementation in the laboratory and point-of-care testing (POCT) in proximity to the patient [15]. To date, electrochemical impedance spectroscopy (EIS) has opened new paths for detecting Tau proteins and has gained auspicious attention due to its undeniable advantages, particularly its ability to quickly identify small perturbations at the electrode/solution interface. Moreover, EIS demonstrates cost-effectiveness and simplicity and does not require any additional reagents. As a result, this platform allows for the concurrent detection of Alzheimer’s biomarkers [16].

Antibody immobilization is a critical procedure in the establishment of impedimetric immunosensors, as the effectiveness of this phase can significantly impact the analytical performance of the immunosensors. In this regard, various immobilization strategies were thoroughly employed, encompassing physical adsorption [17,18], covalent immobilization through self-assembled monolayers (SAMs) [19], electro-polymerization of conducting polymer films [20], electro-grafting of aryl diazonium salts for attachment of an organic film on various electrodes [21], and biotin/avidin interaction [22]. Self-assembled monolayers (SAMs) represent an effective approach for interfacial modification, creating a well-ordered monolayer on a surface via strong chemical bonds, which enhances stability and allows for precise control over surface properties [23]. The use of SAMs has greatly influenced the advancement of electrochemical immunosensors due to their straightforward preparation, robustness, and chemical coupling capabilities. This method presents a more direct approach in comparison to affinity capture and offers enhanced stability over physical adsorption. However, the use of long-chain SAMs is constrained in electrochemical investigations due to their low permeability to electron transfer. This limitation can be addressed by utilizing short-chain SAMs, which enhance efficient electron transfer through the ultra-thin layer defects [24]. In this context, the three-carbon alkane chain cysteamine, an amino-terminated thiol, establishes a covalent bond with gold via chemisorption through the sulfur group, while the amine group is oriented towards the solution. Amino-terminated SAMs demonstrate a significant ability for further modification, with homobifunctional cross-linkers like glutaraldehyde (GA) or 4-phenylene diisothiocyanate (PDITC) utilized to enable the immobilization of the capture antibody to an amine-terminated surface. Glutaraldehyde (GA) is recognized as the leading cross-linking agent, with extensive documentation and utilization throughout its history [25].

The effectiveness is due to the capacity of aldehyde groups in GA to undergo a nucleophilic reaction with the ε-amino group from lysine residues in antibodies, resulting in the formation of Schiff bases (imine bonds) [26]. However, Schiff bases are susceptible to hydrolysis under acidic and neutral conditions, leading to their decomposition and the regeneration of both aldehyde and amine groups. This instability can be addressed by reducing the imine bond with NaBH_4_ or NaBH_3_CN to produce a stable secondary amine linkage [27]. Conversely, the covalent attachment of antibodies to amine-terminated surfaces using the cross-linker p-phenylene diisothiocyanate (PDITC) has recently been employed in the covalent immobilization of proteins for biosensor applications, owing to its capacity to form robust covalent bonds with immobilized biomolecules [28]. This rigid homobifunctional cross-linker has two isothiocyanate groups on its phenyl ring, which are capable of binding to amines. The former interacts with the free amine groups on the cysteamine-functionalized platform to establish a stable thiourea linkage, whereas the latter binds to the ε-amino group from lysine residues in the antibody. Furthermore, PDITC exhibits reduced toxicity and enhanced conductive properties compared to glutaraldehyde (GA) [29].

Despite this, the detection of Tau protein has been improved by electrochemical impedance spectroscopy. The limit of detection continues to pose a considerable challenge, given that the clinical values of Tau protein in real samples are at picogram levels [30]. Additionally, the tiny size of the target Tau protein (40–65 kDa), which is much smaller than the capture antibody, poses inherent challenges for impedimetric biosensing. The key factors influencing the output signal in this system encompass steric hindrance resulting from the immunoreaction between the immobilized antibody and the target analytes, as well as electrostatic repulsion between the free charges of target analytes and the redox couple present in the supporting electrolyte [31,32]. Therefore, it is preferable to employ an amplification approach to boost the sensitivity and improve the limit of detection [33].

To address these challenges, a sandwich-type impedimetric immunosensor is stupendous and has been studied with satisfactory results [34]. For this kind of biosensor, scientists often employ either aptamer [35] or antibodies [36] as the primary bio-recognition element while using the other as a signal amplifier that can be labeled or conjugated to nanomaterials. In this context, our work presented in this manuscript utilizes a dual-antibody sandwich for ultrasensitive Tau-441 detection. The capture antibody HT7, immobilized onto the modified gold electrode, specifically targets the mid-domain epitope (aa 159–163) of human Tau-441 [37]. Following Tau-441 binding, a secondary biotin-labeled detection antibody (BT2-streptavidin complex) recognizes a proximal epitope (aa 194–198) [37], resulting in the formation of a stable immune complex. This dual-epitope system significantly enhances the sensitivity, selectivity, and limit of detection. These results were attributed to the dual-antibody sandwich system through the use of a secondary monoclonal antibody conjugated to streptavidin, and the proposed method has the potential to promote the detection of small-sized antigens and can be applied to the diagnosis of different diseases.

## 2. Materials and Methods

### 2.1. Reagents and Buffers

The recombinant human Tau-441 protein (ab84700, MW 46 kDa) was purchased from Abcam (Shanghai, China). The capture antibody (mAb1, monoclonal, clone HT7, yielded in mouse, MN 1000) was obtained from Thermofisher (Shanghai, China). The capture antibody HT7 specifically binds to the middle domain, encompassing amino acids 159–163, which is common to all isoforms of Tau protein. A secondary biotin-labeled monoclonal detection antibody (mAb2, monoclonal, clone BT2, produced in mouse, MN 1010B) was also purchased from Thermofisher (Shanghai, China) and specifically recognizes the aa-194–198 region of Tau protein. Cysteamine, p-phenylene diisothiocyanate (PDICT), and ethanolamine, used for the functionalization of the gold electrode surface and the immobilization of the HT7, were bought from Sigma-Aldrich (Shanghai, China), along with human serum albumin (HSA) and streptavidin. All solutions were prepared using deionized water. The immobilization of antibodies and antigen-binding were carried out in a phosphate buffer saline solution (10 mM Na_2_HPO_4_, 10 mM NaH_2_PO_4_, NaCl 150 mM, pH 7.4). The detection buffer contains 5 mM K_4_Fe(CN)_6_/K_3_Fe(CN)_6_ dissolved in 10 mM PBS buffer.

### 2.2. Apparatus

The different electrochemical measurements carried out in this work (CV and EIS) were performed by a CHI760E electrochemical workstation purchased from CH Instruments Co. (Shanghai, China). The conventional detection system was composed of a three-electrode configuration, consisting of a disk-type gold electrode with a diameter of 2.0 mm as the working electrode, an Ag/AgCl electrode as a reference electrode, and a platinum wire electrode as a counter electrode. The working, reference, and counter electrodes were bought from CH Instruments Co. (Shanghai, China).

### 2.3. Electrodes Pre-Treatment

The Au electrodes were first polished using alumina powder with a particle size of 1.0, 0.3, and 0.05 μm. Afterward, the electrodes were washed with deionized (DI) water under sonication and then dried with nitrogen gas. After that, the electrodes were incubated for 5 min in piranha’s solution (1:3, *v*/*v*, H_2_O_2_, and H_2_SO_4_), then rinsed with DI water. This was followed by an extra 5 min of sonication in ethanol and drying with nitrogen gas. Finally, the Au electrodes underwent electrochemical cleaning through cyclic voltammetry scanning between 0.20 and 1.6 V vs. Ag/AgCl at a scan rate of 100 mV/s in 0.1 M sulfuric acid until a reproducible voltammogram was obtained [31].

### 2.4. Functionalization of the Au Electrodes

We investigated the self-assembled monolayers (SAMs) system for modifying the gold surface and immobilizing the capture antibody, as described in our previous studies [31,32]. Briefly, the pre-treated electrodes were initially immersed in a 5 mM cysteamine solution for 17 h at room temperature (RT). Subsequently, the cysteamine-modified electrode was rinsed with DI water and high-purity ethanol to remove any adsorbed cysteamine molecules and then dried with nitrogen gas. Consequently, the engendered terminal amino groups were activated by dipping the cycteamine-modified electrode for 1 h in a 10 mM solution of p-phenylene diisothiocyanate (PDITC), which was dissolved in a mixture of pyridine and *N*,*N*-dimethyl formamide (DMF) at a ratio of 1:9 (*v*:*v*), forming a thiocyanate functional group on the cysteamine-modified gold electrode. PDITC was mainly selected for its stability, flexibility, rapid reaction kinetics, and lower toxicity [31]. Afterward, the PDITC-modified gold electrodes underwent rinsing three times with DMF, ethanol, and PBS (pH 7.4). Following this, the functionalized electrode was covered with 50 µL of 20 µg/ mL HT7 antibody in PBS (pH 8.5) for 2 h, followed by washing with PBS to eliminate any unbound antibodies. Next, residual thiocyanate groups were deactivated and unreacted sites blocked by incubating the Au/cys/PDITC/HT7 electrode in 0.1 M ethanolamine (pH 8.5) for 30 min, followed by multiple PBS (pH 8.5) washes. Finally, the HT7-modified electrodes were either immediately employed in subsequent immunosensor experiments or stored in a dry environment at 4 °C for future use.

### 2.5. Characterization of the Immunosensor

The impedance spectra and the cyclic voltammetry were measured at various stages to evaluate and characterize the effectiveness of the Au electrode functionalization and antibody immobilization. The impedance spectra were chronicled over the 100 kHz–0.1 Hz frequency range, with a bias potential of +0.21 V and an AC amplitude of 10 mV. The recorded impedance spectra were displayed in the complex impedance plot (Nyquist plot). A fitting system was employed to analyze the impedance spectra by leveraging a suitable equivalent electrical circuit. The cyclic voltammograms were recorded within a potential range of −0.2 V to 0.6 V. All measurements were conducted in a 10 mM PBS buffer (pH 7.4) in the presence of 5 mM Fe(CN)_6_^3−/4−^ as a redox probe.

### 2.6. Application of the Immunosensor for Tau-441 Detection

All electrochemical measurements were conducted at ambient temperature (~20 °C). In the direct detection format, 50 µL of Tau-441 protein solution, prepared in PBS at different concentrations, was applied to the sensing interface and incubated for 45 min to facilitate specific binding with the immobilized HT7 antibody. After incubation, the electrode was carefully rinsed with PBS to eliminate any unbound Tau-441 molecules. Electrochemical impedance spectroscopy (EIS) measurements were conducted both before and after protein interaction to evaluate changes in surface properties. In the sandwich-type immunoassay configuration, following the binding of Tau-441 to the HT7-modified surface, 50 µL of biotin-labeled secondary antibody (BT2, 10 µg/mL in PBS) was applied to the sensor. This step was conducted with a 45 min incubation period to promote the formation of the antibody-antigen-secondary antibody complex. The generated immuno-complex was successfully washed to remove the unbound biotinylated secondary antibody and then immersed in 5 µg/mL of streptavidin solution. The final immunosensor was thoroughly rinsed with PBS buffer and subsequently subjected to additional impedimetric measurement. The signal was recorded before and after each addition to assess the binding events.

### 2.7. Selectivity, Reproducibility, and Stability

The selectivity test was carried out using 1 ng/mL He4 antigen, Human Albumin Serum, and amyloid-beta proteins. Additionally, the reproducibility test was performed inter-batch (between-batch), and intra-batch (within-batch) was carried out for five, ten, and fifteen days. Moreover, the stability of the designed immunosensor was carried out for two weeks. All the tests were executed in triplicate unless otherwise mentioned.

## 3. Results and Discussion

### 3.1. Sandwich Immunosensors Fabrication

Direct immunosensors are conceptually a simple sensing architecture that enables the conversion of immunoreaction events between analytes and immobilized antibodies into detectable and quantifiable signals. In this particular immunosensor, no additional labels are required for signal amplification, allowing for real-time detection of the analyte and high-throughput analysis. Therefore, the utilization of direct immunosensors in a clinical setting has gained significant popularity in recent decades. However, identifying low-molecular-weight (LMW) proteins with a molecular weight of less than 50 kDa or proteins at very low concentrations in real samples with a direct immunosensors format remains challenging for two reasons: (i) the immunoreaction event between the immobilized antibody and the proteins causes only a small change in the interfacial properties at the sensing surface resulting in a very small analytical signal [38], and (ii) direct immunosensors are subject to a notable impact of non-specific binding (NSB) on their performance [39]. An alternative strategy utilizes the sandwich immunoassay format, in which a primary antibody anchored to a solid substrate selectively captures the target analyte. This is followed by the introduction of a secondary antibody that recognizes and binds to the formed antigen–antibody complex. This method is widely applicable to analytes of different molecular sizes, as long as the target molecule has two distinct and non-overlapping epitopes that enable simultaneous recognition by both the capture and detection antibodies. The process of the analyte detection encompasses a series of sequential steps: (i) immobilization of a capture antibody on the surface of the sensors, (ii) identification of the analyte, and (iii) binding of a labeled detection antibody to the captured analyte, which produces the signal that will be measured. In this case, both the capture and detection antibodies should be able to bind to the analyte simultaneously and independently [40].

**Scheme 1:**
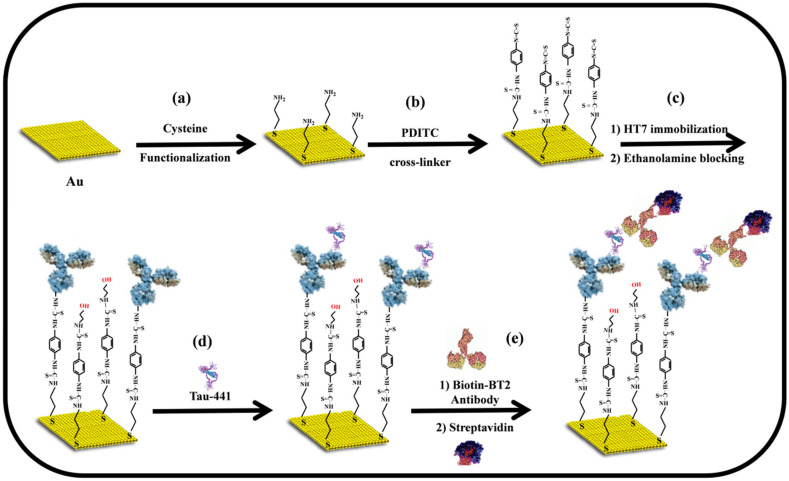
Schematic representation of Tau-441 protein detection employing a sandwich immunosensor. (**a**) Cysteamine assembly on bare gold electrode; (**b**) activation using 1,4 dithiocyanate (PDICT) cross-linker; (**c**) immobilization of the HT7 antibody followed by blocking with ethanolamine; (**d**) immunoreaction between capture antibodies HT7 and Tau-441; (**e**) signal amplification using biotinylated detection antibody (BT2)-streptavidin complex.

In this vein, a signal enhancement technique based on the sandwich format was developed to achieve a high-sensitivity impedimetric immunosensor for the quantification of the Tau-441 biomarker (45 kDa), especially for the determination of a low-concentration target analyte. Scheme 1 illustrates the stepwise procedure for the fabrication of the sandwich-type impedimetric immunosensors for the detection of Tau-441 biomarkers. Capture antibodies (HT7) were covalently immobilized on a cysteamine-modified gold electrode. The process was enabled through the use of PDICT as a cross-linker, where one of the thiocyanate groups reacted with the amine groups present on the cysteamine-modified gold surface, while the other thiocyanate group reacted with the amine groups located on the lysine residue of the antibodies. These reactions resulted in the formation of stable thiourea bonds [41]. A sandwich-type signal amplification approach was employed in the fabrication of this impedimetric immunosensor. Initially, the immobilized capture antibody (HT7), which specifically binds to the mid-domain epitope (amino acids 159–163) of the human Tau-441 protein, was exposed to the protein solution. This resulted in an immunoreaction between the capture antibodies and the protein, leading to the binding of the Tau-441 protein to the sensing electrode. Then, the biotin-labeled detection antibody (BT2), which specifically targets amino acids 194–198 of the Tau-441 protein, interacted in a proportional manner with the bound protein on the sensing electrode. Ultimately, the sandwich complex HT7/Tau-441/BT2 interacted with streptavidin to enhance signal amplification. In this context, an alteration in electrostatic and/or steric barriers at the sensing surface determines the energy barrier involved in the electron transfer process between the redox couple and the electrode surface, which, in turn, affects the faradaic impedance signal, specifically the charge-transfer resistance (R_ct_) [31,32]. As a result, the substantial size and the negative charge of BT2 and streptavidin will lead to a notable enhancement in signal intensity for the impedimetric immunosensor. This enhancement will allow the immunosensor to accurately detect significantly lower concentrations of Tau-441.

### 3.2. Electrochemical Characterization of the Immunosensrs

**Figure 1:**
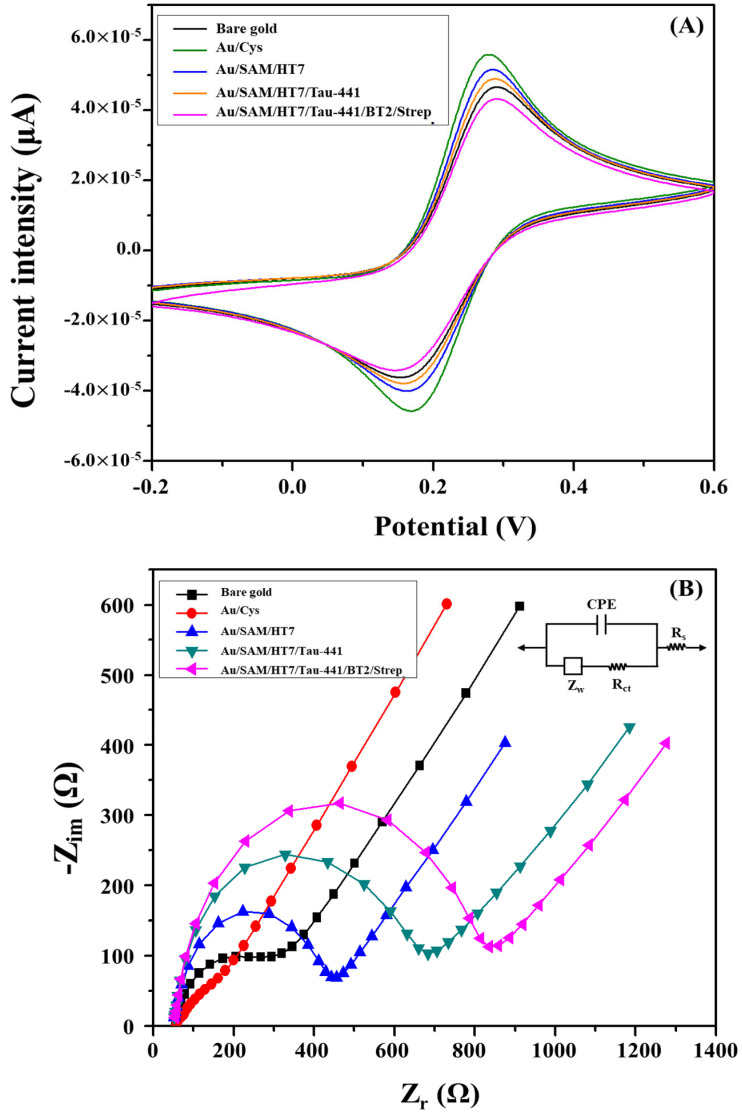
(**A**) Cyclic voltammograms recorded at different steps of the fabrication and detection process in a 10 mM PBS buffer (pH 7.4) containing 5 mM Fe(CN)_6_^3−/4−^ redox couple under a scan rate of 100 mV/s. (**B**) Nyquist diagrams for impedance measurements recorded at different steps of the fabrication and detection process of the proposed immunosensor in the presence of a 5 mM Fe(CN)_6_^3−/4−^ probe dissolved in PBS (pH 7.4) over the frequency range from 10^5^ to 0.1 Hz. The inset is the equivalent Randles circuit.

Cyclic voltammetry (CV) is a very powerful tool for probing the features of a modified electrode surface, providing a rapid and straightforward method for preliminary characterization of the modified electrode using a redox couple system [32]. The CV was performed over a potential range from −0.2 V to 0.6 V with a scan rate of 100 mVs^−1^ in a solution containing 5 mM Fe(CN)_6_^3−/4−^, prepared in 10 mM PBS (pH 7.4). As shown in Figure 1A, progressive changes in both the redox peak positions and current responses were observed as the gold electrode underwent sequential surface modifications, indicating alterations in electron transfer characteristics of the redox probe. At the initial stage, the bare gold electrode displayed well-defined and reversible redox peaks with a peak-to-peak separation (ΔE_p_) of approximately 80 ± 5 mV (Figure 1A, black curve), consistent with a previous study that correlates this behavior with clean and electrochemically active gold surfaces [32]. The formal potential of the redox couple was estimated to be approximately 0.21 V, derived from the mean of anodic and cathodic peak potentials in the cyclic voltammogram of the bare gold electrode. A gold electrode was chemically modified with a monolayer of cysteamine using the well-established gold-thiol (Au-S) chemistry. This led to the formation of NH_2_ groups on the surface of the cysteamine-modified gold electrode. The positively charged amine groups, with a surface pKa of 7.6, were found to be responsible for the electrostatic attraction of the negatively charged redox probe [42]. Consequently, this interaction enabled an enhancement of the interfacial electron-transfer process, yielding a slight increase in peak current and a reduction in peak-to-peak separation to 60 ± 3 mV (Figure 1A, green curve). Following the activation of the amino groups using the PDICT cross-linker, the immobilization of the HT7 antibodies, and the blocking of the surface with ethanolamine, the shape of the CV exhibited a slight alteration, characterized by a reduction in the peak current and an increase in the peak-to-peak separation (Figure 1A, blue curve). This is consistent with the increased electron transfer barriers generated by the assembly of these layers. Specifically, after the antibody is immobilized onto the modified electrode, a significant peak-to-peak separation is observed. This indicates that the immobilization of the antibody insulates the electrode due to its large-size analyte (150 kDa) and being negatively charged (pI 4.65) at the working pH [43], which notably disrupts the interfacial electron transfer.

The electrochemical impedance spectroscopy (EIS) technique was employed to offer additional insights into the interfacial properties of surface-modified electrodes. In comparison to other electrochemical techniques, EIS stands out as an effective and nondestructive technique for investigating the electron-transfer properties of the modified electrodes, unlike the wide potential window used in cyclic voltammetry [44]. Figure 1B shows the impedance spectra depicted as a Nyquist plot for the bare gold electrode (black curve), the cysteamine-modified gold electrode (red curve), and the immobilized HT7 antibody with ethanolamine backfilling (the blue curve) at the formal potential of the Fe(CN)_6_^3−/4−^ redox probe in a 10 mM PBS buffer at pH = 7.4. As a result, the impedance spectrum exhibits a semicircular portion at higher frequencies, which is indicative of the electron transfer-limited process, with a diameter reflecting the charge transfer resistance (R_ct_). Additionally, a linear segment at lower frequencies represents the diffusion-limited electrochemical process. In order to provide more comprehensive information, a modified Randles’ circuit, illustrated in the inset of Figure 1B, was proved to adequately fit the experimental data in this study across the entire frequency range [45]. The fitted curves are presented in Figure 1B (solid lines), indicating a strong correlation between the circuit model and the experimental impedance spectra. In this circuit, R_S_ denotes the solution resistance, R_ct_ indicates the charge-transfer resistance of the Fe(CN)_6_^3−/4−^ redox probe, the constant phase element (CPE) is associated with the double layer capacitance, and the Warburg impedance Z_W_ arises from the diffusion of ions from the bulk electrolyte to the electrode interface. In the context of faradaic impedance spectroscopy, charge-transfer resistance R_ct_ is the most important component among the other electrical components within the equivalent circuit [32]. Upon modification of the bare gold electrode with a cysteamine monolayer, the R_ct_ value decreased notably from 255.6 ± 13.8 Ω (bare gold electrode, black curve) to 156.8 ± 9.4 Ω (cysteamine-modified, red curve), revealing that a monolayer of cysteamine was covalently assembled on the bare gold electrode through Au-S chemistry. This is attributed to the electrostatic attraction force between the positively charged amino groups of cysteamine and the negatively charged redox couple, which enhances the charge transfer process from the redox couple to the electrode surface [32]. After the immobilization of the HT7 antibody on the activated cysteamine-modified electrode using the PDITC cross-linker, and the ethanolamine backfilling introduced steric and/or electrostatic barriers to the interfacial charge transfer, leading to an increase of 364 ± 14 Ω in the R_ct_ value. In conclusion, the results obtained from EIS measurements are consistent with those extracted from the above CV measurements and confirm the successful immobilization of HT7 antibodies on the activated cysteamine-modified gold electrode with the PDICT cross-linker.

### 3.3. Feasibility of the Sandwich-Type Impedimetric Immunosensors

The concentration of Tau-441 protein directly impacts the quantity of biotinylated detection antibodies (BT2) and, therefore, the amount of streptavidin, which subsequently induces changes in the charge-transfer resistance (ΔR_ct_ = R_ct_ − R_ct0_, where R_ct0_ and R_ct_ denote the charge transfer resistance of the immobilized HT7 antibody and subsequent stages of the sandwich immunosensor, respectively). Therefore, to assess the feasibility of the proposed assay for Tau-441 quantification, faradaic impedimetric measurements were conducted on the immobilized HT7 antibodies, in the presence or absence of Tau-441, with or without the biotin-labeled BT2-streptavidin complex. As shown in Figure 1B, the impedimetric signal (green curve) exhibited a slight increase in the presence of 1 ng/mL Tau-441 compared to the background signal (blue curve). Moreover, following the immunoreaction with the biotinylated antibody-streptavidin complex, a further increase in the impedimetric signal (pink curve) was observed in comparison with the background signal. To elucidate the binding procedure between Tau-441 and HT7 as well as the signal amplification, the ΔR_ct_ values before and after signal amplification were examined. As shown in Table 1, Tau-441 (direct format) induced a ΔR_ct_ of 174.3 + 14.6 Ω. This is likely attributable to the formation of a thin insulating layer following the binding of Tau-441 (46 kDa) to the immobilized antibodies (150 kDa), which marginally inhibited the transfer of the Fe(CN)_6_^3−/4−^ redox probe to the gold electrode. This modest rise in R_ct_ demonstrates the limitation of the direct impedimetric immunosensor for the detection of Tau-441. After signal amplification with the biotin-labeled antibody-streptavidin complex (sandwich format), ΔR_ct_ increased to 329.6 ± 16.5 Ω, which can be ascribed to the significant increase in mass and charge relative to biotin-labeled BT2-complex. The binding of the biotin-labeled BT2-streptavidin complex to the Tau-441 causes a much larger change in the insulating barrier, which ultimately leads to an increase in the impedimetric signal and a boost in the sensitivity of the proposed immunosensor. To confirm whether the observed increase in the R_ct_ may partially originate from non-specific binding (NSB) between the immunosensor and the biotin-labeled antibody-streptavidin complex. A control experiment (NC) revealed that the interaction of the complex with immunosensor surface, in the absence of the Tau-441 protein, caused only a minute change of 16.8 ± 5.8 Ω in charge-transfer resistance (R_ct_) (Table 1). This implies that the observed R_ct_ changes did not derive from non-specific binding (NSB), but only from antigen–antibody interactions.

### 3.4. Optimization of the Experimental Conditions

**Figure 2:**
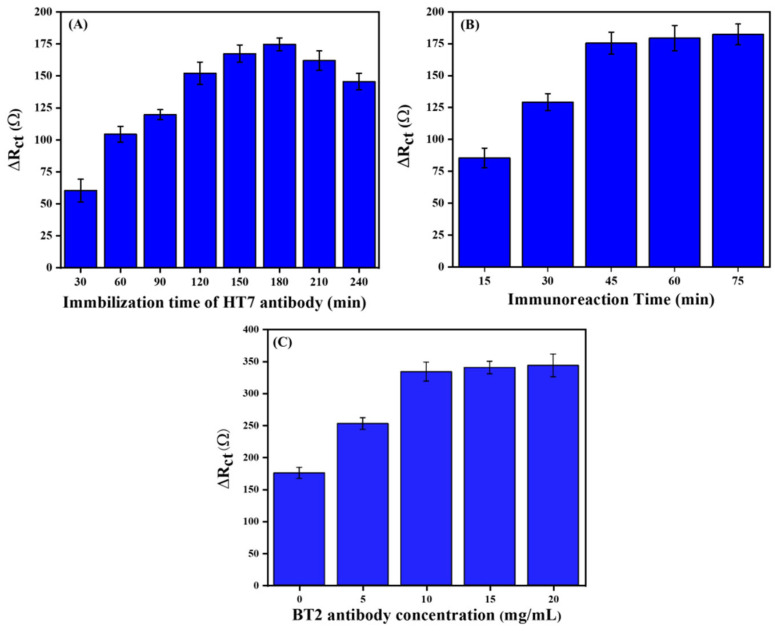
Optimization of (**A**) the immobilization time of HT7 antibody, (**B**) the immunoreaction time, and (**C**) the BT2 antibody concentration, using 10 ng/mL of antigen in 10 mM PBS solution in the presence of 5 mM Fe(CN)_6_^3−/4−^ at pH 7.4.

To achieve optimal analytical performance of our proposed immunosensors, experimental conditions, including HT7 immobilization time, immunoreaction time, and the concentration of BT2 antibody, were optimized for the sandwich-type immunosensor, using faradic electrochemical impedance spectroscopy (F-EIS) in 5 mM Fe(CN)_6_^3−/4−^. The variations in the charge transfer resistance (ΔR_ct_) observed with 1 ng/mL Tau-441 were used to assess the performance of the proposed immunosensor. The sensitivity of the immunosensors depends on the optimal surface density of the immobilized capture antibody (HT7) on the modified gold electrode. For this purpose, the effect of surface density was investigated by incubating 1 μg/mL of HT7 onto a PDICT-modified gold electrode over a range of different periods (30–240 min) at pH 7.4 with the immunoreaction time fixed at 45 min. As depicted in Figure 2A, it is evident that the response of ΔR_ct_ gradually rises with the increase in incubation time of HT7, ranging from 30 to 180 min, reaching a maximum at 180 min. Nevertheless, upon extending the incubation time of the HT7, a decline in the response of ΔR_ct_ was observed. This observation aligns with the hypothesis that an excessively high surface density of immobilized antibodies can impede the binding event due to steric hindrance [46]. Hence, the duration of 180 min has been chosen as the incubation time for HT7 to proceed with further experiments. In addition, the immunoreaction time is a crucial parameter that has a significant impact on the analytical performance of immunosensors. In this context, the effect of immunoreaction time on the impedimetric immunosensor was examined between 15 and 75 min. The result revealed (Figure 2B) that the ΔR_ct_ response increased with increasing immunoreaction time and reached a plateau after 45 min, indicating that the binding event between Tau-441 and immobilized HT7 had occurred. Thus, for the subsequent experiments, the chosen immunoreaction time was 45 min. Finally, the effect of BT2 antibody’s concentration was investigated, ranging from 0, 5, 10, 15, and 20 µg/mL with a fixed concentration (5 µg/mL) of streptavidin. As shown in Figure 2C, the signal gradually increases from 0 to 10 µg/mL, after which it stabilizes as a result of saturation. The optimal concentration of the BT2 antibody was selected as 10 µg/mL.

### 3.5. Dose Response of Sandwich Immunosensors Platform Toward Target Tau-441

**Figure 3:**
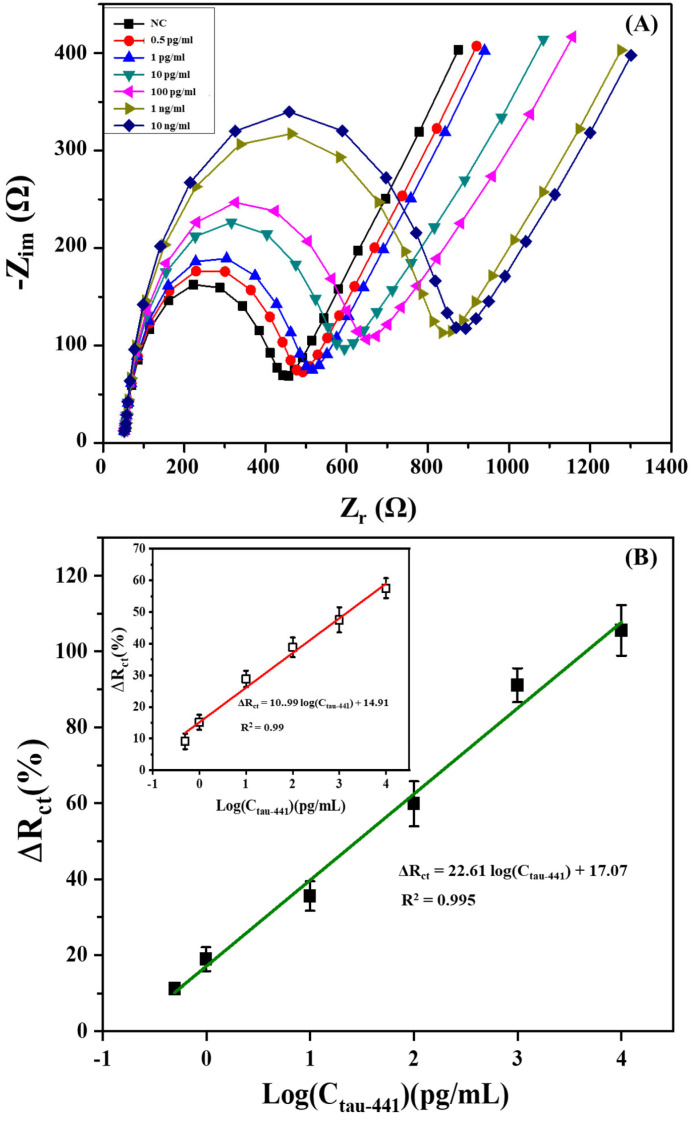
(**A**) Nyquist diagram (Z_r_ vs. Z_im_) for sandwich-based immunosensor in the presence of the Tau-441 under a range from 0.5 pg/mL to 10 ng/mL in a 10 mM PBS solution in the presence of 5 mM Fe(CN)_6_^3−/4−^ at pH 7.4. (**B**) The calibration plot related to the increase of relative electron-transfer resistance (∆R_ct_ (%)) of the sandwich-based immunosensor with different Tau-441 concentrations in PBS, respectively. The inset is the calibration curve of the direct immunosensor. Error bars reveal the standard deviation of three distinct repetitive measurements.

The sandwich-type impedimetric immunosensors were effectively used for the detection of Tau-441 under the optimized conditions. The impedimetric measurement was monitored using various concentrations of Tau-441, ranging from 0.5 pg/mL to 10 ng/mL. According to the data presented in Figure 3A, it can be observed that the diameter of the semicircle (R_ct_) gradually increased as Tau-441 concentrations increased. The observed phenomenon can be mainly attributed to the sandwich immunological binding event. This event creates additional steric and/or electrostatic barriers that hinder the interfacial charge transfer between the Fe(CN)_6_^3−/4−^ redox couple and the electrode surface. As a result, the R_ct_ value increased. In order to conduct the impedance analysis thoroughly, the acquired findings were fitted using the suggested modified Randle’s equivalent circuit (shown in the inset of Figure 1B). As anticipated, a notable variation in the charge transfer resistance (R_ct_) was observed when the concentration of Tau-441 increased. In contrast, the variations in R_s_, CPE, and Z_w_ were minimal and inconsistent [32]. These findings provide evidence for the efficacy of R_ct_ as a quantitative parameter for assessing the analytical capabilities of the sandwich-type impedimetric immunosensor. To avoid electrode-to-electrode variations, we plotted the normalized response (ΔR_ct_ (%) = (R_ct_ − R_ct0_)/R_ct0_) against the concentration of Tau-441, where R_ct0_ and R_ct_ represent the charge transfer resistance of the immobilized HT7 antibody and after the sandwich immunological binding event for a given amount of Tau-441. As depicted in Figure 3B, the calibration plot exhibited a good linear relationship between the normalized response ΔR_ct_ (%) and the logarithm concentration of Tau-441 in the range from 0.5 pg/mL to 10 ng/mL. The regression equation of this calibration curve was ΔR_ct_ (%) = 22.61 log(C_Tau-441_) + 17.07 with a correlation coefficient of 0.995, where c was the concentration of Tau-441 (pg/mL). The limit of detection (LoD) of this proposed immunosensor, defined as the Tau-441 concentration at which the signal is three times the standard deviation above zero calibrator (n = 3) [47,48], was approximately 0.08 pg/mL. To assess amplification efficiency, impedimetric measurements were conducted in the direct format using identical conditions. In this case, the regression equation of this calibration curve was ΔR_ct_(%) = 10.99 log(C_Tau-441_) + 14.91 (R^2^ = 0.99) in the range of 1 pg/mL to 10 ng/mL, with a LoD of 0.9 pg/mL, which is approximately 11 times higher than the limit of detection achieved through the sandwich format (shown in the inset of Figure 3B). Hence, the use of a biotin-labeled antibody-streptavidin complex, possessing higher molecular weight and charge in comparison to Tau-441, resulted in the enhancement of the engineered immunosensor. Significantly, in clinical practice, a level of 350 pg mL^−1^ for Tau-441 in human CSF samples is considered the cut-off value for the potential diagnosis of Alzheimer’s disease [49]. The sandwich immunosensor described in this study effectively quantifies Tau-441 biomarkers, enabling the differentiation of AD patients from other individuals.

For the sake of comparison with different detection methods, Table 2 presents a summary of the performance parameters associated with various electrochemical methods and commercial ELISA kits. The analytical performance achieved with the proposed sandwich immunosensor for Tau-441 detection significantly exceeds that of the commercial ELISA kits reported in the literature. For instance, the INNO-BIA AlzBio3 and the INNOTEST plate ELISAs have been specifically designed for the detection of Tau protein. Albright’s group conducted a study on these kits, experimenting with different antibody combinations. Notably, the HT7 and BT2 antibodies were highlighted, indicating that the highest CSF Tau levels were identified using the HT7-BT2 assay, which had a limit of quantification of 7.8 pg/mL [37]. However, ELISA exhibited certain limitations such as low sensitivity, narrow dynamic range, and high cross-reactivity. Moreover, compared to other previous studies, this study revealed a comparable or an outperformed limit of detection. For instance, Karaboga and Sezgintürk [16] established an impedimetric biosensor using nanocomposites consisting of reduced graphene oxide (rGO) and gold nanoparticles (AuNP) for Tau-441 detection, and they were able to detect as low as 0.091 pg/mL. However, this method was limited by single-use and potential variability in nanocomposite synthesis. In another report, a better limit of detection was achieved in the range of 2.3 fg/mL using MXene-MIP Redox System [50]. Yet, a complex fabrication system and reproducibility issues limited this technique. Therefore, the procedure of fabricating the immunosensor in this investigation is characterized by its simplicity and lack of requirement for further enzyme amplification. Consequently, this approach offers a time-efficient, cost-effective, and remarkably sensitive device for detecting Tau-441, and it may potentially serve as an innovative tool for widespread screening of Alzheimer’s disease.

### 3.6. Selectivity, Reproducibility, and Stability of the Developed Sandwich Immunosensor

**Figure 4:**
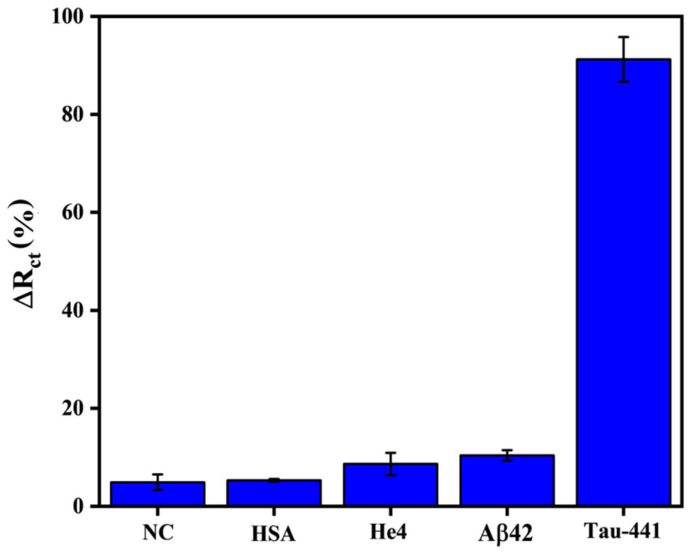
Comparative histograms illustrating the ∆R (%) of the Tau-441target (1 ng/mL) alongside off-target proteins (1 ng/mL) using the proposed sandwich immunosensor.

In order to conduct a more comprehensive analysis of the impedimetric sandwich-type immunosensors, a number of tests were conducted to assess their specificity, repeatability, and stability. The selectivity of the immunosensor is widely recognized as a critical factor that can significantly impact its overall performance. In this context, three proteins, namely HSA, He4, and beta-amyloid-42 (Aβ42), have been selected as interfering proteins, each with different molecular weights and isoelectric points (pI). HSA represents the principal protein fractions found in serum, possessing a molecular weight of around 67 kDa. Similar to Tau-441, beta-amyloid-42 is likewise recognized as a neurodegenerative biomarker in CSF and serum, possessing a molecular weight of 2.4 kDa. The protein known as He4, which is frequently employed as a biomarker for tumors, exhibits a molecular weight that is comparable to that of Tau-441. The sandwich immunosensor was subjected to a typical experiment in which it was challenged with 1 ng/mL of these proteins under identical experimental circumstances, and the evaluation was carried out by comparing their normalized signals. As displayed in Figure 4, a clear normalized signal was observed for Tau-441, whereas weak normalized signals were observed for the off-target proteins and the negative control (NC—the modified gold electrode with anti-HT7 exposed to PBS solution without Tau-441 protein for 45 min), which are approximately 10 times lower than the normalized response of Tau-441, denoting that there was no considerable cross-interaction between the designed immunoassay and the interfering proteins. These results provide fervent evidence that the recommended immunosensor possesses a high selectivity toward the detection of Tau-441. This observation can be elucidated by the advantages of our sandwich assay, which offers great specificity and sensitivity due to the utilization of two matching antibodies (HT7 and BT2).

The reproducibility of the developed immunosensor was assessed by testing its performance with a 1 ng/mL solution of Tau-441 protein. Both intra-assay and inter-assay variations were evaluated. In the intra-assay assessment, three parallel measurements were performed under identical conditions, resulting in a relative standard deviation (RSD) of 7%. The inter-assay reproducibility was assessed through the implementation of three independent assays using different immunosensor batches prepared under identical experimental conditions, yielding an RSD of 10%. The findings demonstrate that the proposed immunosensor shows satisfactory reproducibility and reliability for the quantitative detection of Tau-441.

To investigate the stability of the proposed immunosensor, the modified electrodes with HT7 antibody were stored at 4 °C in PBS before testing, and the output signal was then recorded every five days after being treated with 1 ng/mL Tau-441 protein. The stability test results of the immunosensor demonstrated consistent performance over a two-week period, indicating its robustness and reliability for practical applications. The proposed immunosensor maintained over 85 ± 4% of its initial response after 14 days of storage at 4 °C in PBS, suggesting excellent long-term stability. These findings validate the immunosensor’s suitability for routine diagnostic use and highlight its potential for integration into point-of-care testing systems.

## 4. Conclusions

Tau protein represents a promising biomarker for the early diagnosis of Alzheimer’s disease. Recently, numerous techniques have been established for the detection of this protein. Electrochemical biosensors serve as cost-effective and efficient analytical tools for quickly testing a selected target, ensuring high specificity and sensitivity. To date, several biosensors for the detection of Tau protein have been reported using different approaches. Despite advancements in the protein detection process, the LoD remains a challenge, as clinical values of Tau protein in CSF are in the pico-gram level. In this work, we developed a sandwich-type immunosensor, wherein we investigated an amplification strategy. This proposed sandwich assay demonstrated an improved limit of detection (LoD) in the range of 0.08 pg/mL, in contrast to the direct approach, which had an LoD in the range of 0.9 pg/mL. These findings were attributed to the enhancement of the normalized signal through the use of a secondary monoclonal antibody conjugated to streptavidin. Additionally, the application of the developed immunosensor in the presence of various off-targets yielded no cross-reactivity. As a result, our proposed method has the potential to enhance the detection of small-sized antigens and can be applied to the diagnosis of different diseases.

## Figures and Tables

**Table 1:** Fitted values of the charge transfer resistance (R_ct_) derived from the modified Randle circuit. Highlighted the standard deviation (SD) using red font.

	R_ct_ (Ω)	ΔR_ct_ (Ω)
Au/SAM/anti-HT7	364	--
Au/SAM/anti-HT7/Tau-441 (1 ng/mL)	538.3	174.3 ± 14.6
Au/SAM/anti-HT7/Tau-441/BT2-Strep	693.6	329.6 ± 16.5
NC (Sandwich format without Tau-441)	380.8	16.8 ± 5.8

**Table 2:** Performance comparison of Tau-441 detection based on various methods and materials used.

Detection Method	Modified Electrode	Linear Range	LOD/LOQ	Refs.
Sandwich type ELISA (HT7-BT2)		10–100 pg/mL	7.8 pmol/L	[18]
SWV	SPCE/PBNCs/GO-MIP	1.09–2.18 nmol/L	0.01 pmol/L	[51]
EIS	ITO/rGO-AuNP	1–500 pg/mL	0.091 pg/mL	[16]
DPV	WE/PEDOT:PSS/MWCNTs	0.00001–50 μg/mL	7.4 pg/mL	[52]
DPV	Au/MWCNTs-rGO-CS	0.023–3.672 pg/mL	0.021 pg/mL	[53]
DPV	SPGE/VxPDA-MIP	5 fg/mL–5 ng/mL	2.3 fg/mL	[50]
EIS	Au/Cyst-PDICT	0.5 pg/mL–10 ng/mL	0.08 pg/mL	This work

## Data Availability

The datasets used during the current work are available from the corresponding authors upon reasonable request. The data are not publicly available because they are part of an ongoing study.
